# Re-thinking discourses of “youth” within (adult) regulation of skateboarding

**DOI:** 10.3389/fspor.2024.1473992

**Published:** 2024-10-21

**Authors:** Robert Petrone

**Affiliations:** Department of Learning, Teaching, and Curriculum, University of Missouri, Columbia, United States

**Keywords:** youth & adolescence, youth, skateboarding, regulation, youth as metaphor, neocolonialism

## Abstract

Situated within the context of increasing “adult regulatory practices” in skateboarding, this article draws attention to and interrogates the ways normative ideas of “youth,” “adolescence,” and “youth/adolescent development” often interplay with such efforts. In doing so, this article offers a critique, from a Critical Youth Studies perspective, of dominant, developmentalist notions of youth that typically cast young people as deficits in need of specific forms of intervention and surveillance. The article concludes with areas of inquiry emergent from critiques of dominant renderings of youth to be considered when engaging in processes of forming regulatory programs, policies, and practices related to skateboarding.

## Introduction

As skateboarding has become a mainstream, worldwide endeavor, there have been a surge of policies, programs, and practices related to the management of skateboarding. This regulation has heightened in recent years, as an ever-widening range of stakeholders—from municipalities to parent groups to NGOs to private corporations—have become increasingly involved in skateboarding (1). As a researcher at the intersection of youth studies, skateboarding, and education, I have become increasingly curious about such regulatory practices, especially how the concepts “youth” and “development” get mobilized.

On the one hand, I recognize such oversight as generative for access, inclusion, and diversity—all of which have been long-standing concerns in skateboarding (2, 3). For instance, for many years, skateboarding was dominated by cisgender, heteronormative young (white) men and masculine codes (4). Within this milieu, female, women-identified, and LGBTQ + participants have sometimes been excluded, relegated to the margins of participation, and/or “othered” in demeaning ways (5, 6). In fact, in their discussion of sexual violence in skateboarding, Willing and Pappalardo (7) argue that the *lack of regulation* in skateboarding is partly responsible for these activities (p. 208–209).

Interventions spearheaded by “change maker” skateboarders (7) and other entities have increasingly opened access for female, women-identified, and queer participants. For instance, the organization Skate Like a Girl illustrates how targeted efforts to draw attention to gendered dynamics within skateboarding can lead to increased and enhanced participation for female, women-identified, and queer skateboarders. Such advancements are undeniable in how they facilitate access and raise consciousness about power dynamics in and beyond skateboarding. Furthermore, such initiatives offer powerful testaments to the potential for social transformation offered by official regulation.

At the same time, I wonder about the ways such oversight may also have unintended, adverse effects. Related to the suspect ways neoliberal ideologies often shape regulatory practices, especially related to skateparks (1, 8), I wonder about how some of these overt regulatory moves may, ironically, compromise key facets of learning, participation, and so-called youth development.

Consistent with other scholarship focused on action sports and learning (9), my research (10) demonstrates that engagement in skateboarding is often propelled by its participant-driven ethos. This dynamic is typically noted as central to not only individualized experiences of autonomy and agency but also communal commitments and experiences of belonging. For example, at the skatepark I studied, the participants—because the park was mostly “unregulated”—developed roles as educators and contributors, which cultivated emotional bonds, distributed problem-solving, and a sense of responsibility to the community. Moreover, participants developed self-directed learning curricula, which facilitated both their learning how to skateboard and their understandings of learning as a sociocultural process. In these ways, a lack of regulation engendered rich opportunities for participation, learning, development, and identity formation—that positive youth development occurred “not in spite of, but because of the lack of strict rules, formal leaders or *a priori* performance goals” [(11), p. 1990]. [This is not to say, of course, regulation does not also produce positive impacts; see, for example, Sorsdahl et al. (12), on mental health benefits from a skateboard program.]

And yet, I recognize that unregulated spaces privilege participants whose identities most closely align with dominant social structures. I wonder, then, particularly considering increased regulation in skateboarding and my interests in youth development, about this tension between the best of a participant-driven ethos, often made available from a lack of external regulation, and the ways external regulation disrupts oppressive systems, and, as such, engenders inclusion for a wider swath of skateboarders. To be clear, I am not advocating for a return to some idealized “good old days”—I’m definitely *not* suggesting we need to “make skateboarding great again.” Moreover, my aim here is not to settle these tensions but rather to stimulate inquiries to support them but with more attention to critical perspectives of youth and youth development.

Thus situated, in the remainder of this essay, I critique how normative ideas of “youth” and “adolescence/ts” often undergird and provide rationale for regulatory practices that increasingly shape skateboarding. Throughout my discussion, I draw on Atencio et al. (1), whose multi-sited research in the U.S. demonstrates ways neoliberalism and corporate interests commingle, along with other stakeholders, to engender certain “adult regulatory practices” reliant upon particular ideas of “youth.” In doing so, my hope is to offer possibilities for rethinking how notions of “youth,” “adolescence,” and “development” might factor into rationales regarding management of skateboarding. Although situated within the U.S., at the end of the next section, I offer ways this inquiry might support efforts regarding regulation in other contexts.

### Rethinking normative notions of “youth”

Privatization often operates in sync with youth development agendas based in the urban core…In fact, scholars have already noted that youth are generally considered by adults as being “difficult, deficient, and at risk.” This has often meant that the urban space is “produced as an adult space,” which engenders specific consequences for youth as they are often considered a detrimental presence. And youth have accordingly been subjected to adult regulatory mechanisms, including surveillance and restrictions. Practices to control “undesirable ‘others’—notably youth,” have been especially prevalent under privatization efforts intended to “revitalise or aestheticise” urban space.

Atencio et al., Moving Boarders, 2018, p. 50

In their study of four urban skateparks in the U.S., Atencio et al. (1) explore how neoliberalism structures youth participation in skateboarding. Through their analysis, they show that skateboarding has become an important site whereby interests across myriad stakeholders and ideologies often commingle and constitute one another—from neoliberalism's push toward privatization to municipalities’ aims of gentrification to parents’ hopes for their children to acquire social capital. Frequently, the ostensible common denominator across stakeholders is “youth” and “youth development.” As they explain, “parents and other adults representing corporate, government, and social-activist interests consistently understood urban skateboarding could be used as a new developmental tool for youth” (p. 218–219).

From a Critical Youth Studies (CYS) perspective [e.g. (13)], it is imperative to interrogate underlying assumptions and ideas of “youth” (and proxies like “adolescence/ts” and “teenagers”) that undergird and provide rationale for decision-making and interventions related to this demographic. CYS scholarship demonstrates how people labeled “youth” and “adolescents” are theoretically conceptualized significantly affects the ways practices, programs, and policies targeting this demographic are developed and implemented [e.g. (14, 15)]. For instance, in a comparison of young people's experience of similar school systems in Tibet and Germany, Cribari-Assali (16) demonstrates that differences in how students were viewed and treated by adults accounted for differentiated experiences across these cultural groups. In other words, conceptualizations of youth have consequences for how said youth will be treated, advocated for, punished, etc.

Typically, ideas of youth that undergird adult regulatory practices draw on developmental psychology, and, as such, developmentalism operates as a dominant, authorizing discourse when it comes to forming policies in relation to people labeled “youth,” “teenagers,” “adolescents.” As Burman (15) explains, “developmental psychology exercises a powerful impact on everyday lives and ways of thinking about ourselves” (p. 2). Such framing, particularly as it usually locates the experience of youth primarily in the body and mind and mobilizes discourses like the “teenage brain,” often get understood as “natural” and “universal”—to the point where the “effects are such that they are often almost imperceptible, taken-for-granted features about our expectations of ourselves, others, and parents, children and families, informing the structure of popular and consumer culture as well as explicit technical, official policies” (p. 2). This essentialized, commonsensical conceptualization of youth is what makes permissible diminishing renderings like the following, which appeared in the *New Yorker*: “Adolescents in 2015 can find partners by swiping right on Tinder; nevertheless, they retain the neurophysiology of apes (and, to a certain extent, mice). Teenagers are, in this sense, still swinging through the rain forest, even when they’re speeding along in a Tundra.” [(17), Aug 24].

Offering important critiques to these normalized renderings of youth, CYS recognizes that what has become known as the natural life stage of “adolescence” or “youth” are not universally-experienced, scientifically-verifiable truths but rather social and historically constructed entities—cultural constructs much like other categories of representation like gender. Ideas of youth are socially produced, and thus, always constituted through social arrangements and systems of reasoning within specific contexts at particular moments.

Within the U.S. context, the so-called universal story of youth is—and has been since its inception—rooted in whiteness, masculinity, and nationalism. Lesko (18) explains how adolescence emerged in the U.S. in response to massive shifts in the social order (e.g., mass immigration) that ushered in and/or exacerbated anxieties linked with entrenched (white male) power regarding whiteness, masculinity, and national identity. In this way, adolescence functioned as a “social space in which to talk about the characteristics of people in modernity, to worry about the possibilities of these social changes, and to establish policies and programs that would help create the modern social order and citizenry” (p. 5). In other words, adolescence emerged (and continues to function) as discourse surrogate as it has enabled people (in power) to talk about things like national identity or race without having to talk directly about these things; instead, they could talk about “adolescence/ts.”

From adolescence, normative ideas of “development” soon became codified, and a host of constitutive programs, policies, and practices cropped up, including the Boy Scouts of America and the Playground Association of America. These entities were designed to manage, and direct “proper” developmental trajectories. As Howell (8), in his discussion of skateparks as neoliberal playgrounds, explains: “If there was broad agreement that children proceeded through developmental stages, there was also broad agreement that their development must be directed, that there was a ‘normal course of play’ that would not unfold properly without social influence from trained adults and from peers who were developing normally.” (p. 489). In these ways, adolescence and adolescent development become institutionalized and naturalized.

Interdisciplinary research over the past several decades has demonstrated how normalized ideas of adolescence/ts produce differentiated, adverse outcomes for BIPOC youth and youth who, due to aspects of their intersectional identities (e.g., sexuality), do not fit the expectations of normative adolescence [e.g. (19, 20)]. This scholarship reveals how adolescence and adolescent development in the U.S. uphold dominant social systems like white supremacy and settler colonialism. For this reason, when initiatives regarding regulation in skateboarding conjure ideas of “youth (development),” it is vital they be interrogated.

It is important to note that, though my critique is situated in the U.S., my hope is this inquiry might support efforts to establish nuanced skateboarding regulatory practices in other contexts. As one reviewer of this article asked, “How is the US context relevant and helpful to the rest of the world?” Though not able to fully explore this topic here, it is critical and timely, particularly given the exponential global growth of skateboarding. Furthermore, notions of “youth” and “development” operating as symbolic placeholders are not, of course, exclusive to the U.S.; in fact, examination of such discourses may have even more significance in places often deemed in deficit ways like “developing” and “Third World,” given how discourses of “youth,” “development,” and “nation-building” so often get fused and provide justification for myriad (inter)national policies and practices (21).

Further, because a significant portion of the growth of worldwide skateboarding is due to NGOs and other entities located in the U.S. and, more broadly, the Global North—many of which center ideas of “children,” “youth,” and “development”—such a configuration begs questions of how skateboarding could potentially be a manifestation of concerns raised by many in the Global South, as well as recent critiques of movements such as Sports for Peace and Development, which some argue operates as a form of “neocolonialism” [e.g. (22, 23)]. Many Global South child/youth studies scholars have critiqued the ways Global North discourses of “childhood” and “youth” often frame these ideas in/for the Global South and displace theoretical frameworks more consonant with Global South contexts, leading to “onto-epistemological imbalance” in scholarship, policies, and programs in the Global South (14, 24).

I draw attention to this, especially, given the ways the U.S. is often positioned and positions itself in paternalistic ways. Paying attention to how discourses of “youth” and “development” get mobilized within the U.S. regarding skateboarding, then, might also create opportunities for more nuanced critiques of how similar discourses get mobilized elsewhere to better ensure that local onto-epistemological frameworks govern the management of skateboarding in these contexts rather than those imposed by the U.S and Global North.

### “Youth” & (adult) regulatory practices in skateboarding

All regulatory processes, practices, and programs—whether related to schools or skateboarding—operate from theories of people, in this case “youth,” regardless of the extent to which these are made explicit. And because interpretations of youth profoundly shape opportunities available for the people the policies are ostensibly for, it is necessary to locate, make explicit, and interrogate the discourses circulating that justify regulatory processes.

From this perspective of youth, a host of questions become potentially generative in developing regulatory practices regarding skateboarding:

• In what ways do ideas of youth, adolescence, and development get implored by stakeholders to justify and provide rationales for regulatory practices for skateboarders?
• What theories of youth and development are the regulatory practices operating from?
• How are ideas of youth (development) functioning symbolically as proxies for broader initiatives (whether overtly named or not), including, potentially, other countries, NGOs, and/or for-profit corporations?
• In what ways do discourses of nationhood/building, gender, sexuality, socioeconomics, race, class, and/or ability intertwine with discourses of youth?
• In rationales for regulation, what relationships are made between individual youth development and broader community, city, economic, or national development?
• How do conceptions of youth differ across stakeholders? To what extent are these differences representing youth as a decontextualized, naturalized experience vs. one that recognizes entangled social systems and power relations?
• In what ways are local, context-specific, and/or Indigenous conceptions of youth and development being drawn on?
• To what extent do members of this demographic, one typically removed from official political processes, have a true voice in shaping the policies aimed at them?

By laying out these areas of inquiry and the broader critique my hope is to infuse regulatory efforts with additional ways to (re-)consider conceptualizations of youth. The underlying concern is that youth often become a site for social engineering of typically unspoken interests. And, as Austin and Willard (25) urge: “Youth and young people must be understood as more than longstanding metaphors for adult agendas, desires, or anxieties” (p. 2). This inclination seems especially pertinent in skateboarding given the ways it has, since its inception and perhaps due to its unregulated nature, engendered opportunities for young people to exist beyond and defy normative, deficit renderings of “youth.”

## Data Availability

The original contributions presented in the study are included in the article/Supplementary Material, further inquiries can be directed to the corresponding author/s.

## References

[1] Atencio M Beal B Wright EM McClain Z. Moving Boarders: Skateboarding and the Changing Landscape of Urban Youth Sports. Fayetteville, AK: University of Arkansas Press (2018).

[2] Sueyoshi A. Skate and create: skateboarding, Asian pacific America, and masculinity. Amerasia J. (2015) 41(2):2–23. 10.17953/aj.41.2.2

[3] Williams N. Understanding race in skateboarding: a retrospection and agenda for the importance of being seen. In: Dupont T Beal B, editors. Lifestyle Sports and Identities: Subcultural Careers Through the Life Course. New York: Routledge (2022), p. 284–96.

[4] Carr J. Skateboarding in dude space: the roles of sports and space in constructing gender among adult skateboarders. Sociol Sport. (2017) 1(34):1–30. 10.1123/ssj.2016-0044

[5] Dobija-Nootens N. Understanding the Neal Hendrix Allegations and Power Dynamics in Skateboarding. Brooklyn, New York: Jenkem Magazine (2018).

[6] Rannikkoa A Harinena P Torvinena P Liikanenb V. The social bordering of lifestyle sports: inclusive principles, exclusive reality. J Youth Stud. (2016) 19(8):1093–109. 10.1080/13676261.2016.1145640

[7] Willing I Pappalardo A. Skateboarding, Power, and Change. Palgrave Singapore: Macmillan (2023).

[8] Howell O. Skatepark as neoliberal playground: urban governance, recreation space, and the cultivation of personal responsibility. Space Cult. (2008) 11(4):475–96. 10.1177/1206331208320488

[9] Ellmer E Rynne S Enright E. Learning in action sports: a scoping review. Eur Phy Educ Rev. (2020) 26(1):263–83. 10.1177/1356336X19851535

[10] Petrone R. Dropping In: What Skateboarders Can Teach Us about Learning, Schooling, and Youth Development. Amherst, MA: University of Massachusetts Press (2023).

[11] Säfvenbom R Wheaton B Agans JP. ‘How can you enjoy sports if you are under control by others?’ Self-organized lifestyle sports and youth development. Sport Soc. (2018) 21(12):1990–2009. 10.1080/17430437.2018.1472242

[12] Sorsdahl K Davies T Jensel C Oberholzer D Gelberg L van der Westhuizen C. Experiences and benefits of a youth skateboarding program in South Africa: from the physical to the emotional and beyond. J Adolesc Res. (2021) 39(3):770–95. 10.1177/07435584211052983

[13] Ibrahim A Steinberg SR. Critical Youth Studies Reader. New York: Peter Lang (2014).

[14] Abebe T Dar A Lyså IM. Southern theories and decolonial childhood studies. Childhood. (2022) 29(3):255–75. 10.1177/09075682221111690

[15] Burman E. Deconstructing Developmental Psychology. New York: Routledge (2017).

[16] Cribari-Assali C. Children’s resilience and constructions of childhood: cross-cultural considerations. In: Twum-Danso Imoh A Bourdillon M Meichsner S, editors. Global Childhoods Beyond the North-South Divide. Cham: Palgrave Macmillan (2019) p. 165–86.

[17] Kolbert E. The Terrible Teens. New York: The New Yorker (2015).

[18] Lesko N. Act Your age! A Cultural Construction of Adolescence. New York: Routledge (2012).

[19] Bettie J. Women Without Class: Girls, Race, and Identity. Oakland, CA: University of California Press (2014).

[20] Johnston-Goodstar K. Decolonizing youth development: re-imagining youthwork for indigenous youth futures. AlterNative. (2020) 16(4):1–9. 10.1177/1177180120967998

[21] Burman E. Developments: Child, Image, Nation. New York: Routledge (2021).

[22] Darnell SC Hayhurst LMC. Sport for decolonization: exploring a new praxis of sport for development. Progress Develop Stud. (2011) 11(3):183–96. 10.1177/146499341001100301

[23] Peachey JW Musser A Shin NR Cohen A. Interrogating the motivations of sport for development and peace practitioners. Int Rev Sociol Sport. (2018) 53(7):767–87. 10.1177/1012690216686856

[24] Swartz S Cooper A Batan C Causa LK. The Oxford Handbook of Global South Youth Studies. Oxford, UK: Oxford University Press (2021).

[25] Austin J Willard MN. Generations of Youth: Youth Cultures and History in Twentieth-Century America. New York: NYU Press (1998).

